# Laparoscopic Management of a Very Rare Case: Cystic Artery Pseudoaneurysm Secondary to Acute Cholecystitis

**DOI:** 10.1155/2016/1489013

**Published:** 2016-08-22

**Authors:** Deniz Alis, Sina Ferahman, Süleyman Demiryas, Cesur Samanci, Fethi Emre Ustabasioglu

**Affiliations:** ^1^Department of Radiology, Cerrahpasa Medical Faculty, Istanbul University, 34098 Istanbul, Turkey; ^2^Department of General Surgery, Cerrahpasa Medical Faculty, Istanbul University, 34098 Istanbul, Turkey

## Abstract

Pseudoaneurysm of a cystic artery is a rare entity that commonly occurs secondary to biliary procedures. Most of the cases in literature are consisted of ruptured aneurysms and to our knowledge, except our case, there were only 3 cases with unruptured aneurysms, which incidentally were detected by radiological methods. When cystic artery pseudoaneurysm is present with acute cholecystitis, most of the reports in literature suggested open cholecystectomy with the ligation of the cystic artery as a main treatment option. In this paper we present a case of acute cholecystitis with unruptured cystic artery pseudoaneurysm that incidentally was detected by computed tomography (CT). Cystic artery pseudoaneurysm was handled laparoscopically with simultaneous cholecystectomy. Due to high risk of rupture, surgeons have evaded laparoscopic approach to acute cholecystitis, which accompanied cystic artery pseudoaneurysm. However herein, we proved that laparoscopic management of cystic artery pseudoaneurysm with simultaneous cholecystectomy is feasible and reliable method.

## 1. Introduction

Pseudoaneurysm of the cystic artery is a very rare entity. Majority of the cases are complications of biliary procedures [1]. Acute or chronic cholecystitis and cholelithiasis are uncommon causes of cystic artery pseudoaneurysms [2]. Most of the cases in the literature consisted of ruptured aneurysms. Unruptured pseudoaneurysm that is incidentally detected during radiological examinations is so rare, and by our knowledge there are only 3 cases identified in the current literature [3]. In this paper we report a case of unruptured cystic artery aneurysms accompanying acute cholecystitis, which was detected during radiological examinations and was handled laparoscopically with simultaneous cholecystectomy. By our knowledge, there is only 2 reports of this in English literature [2].

## 2. Case Report

**Figure 1:**
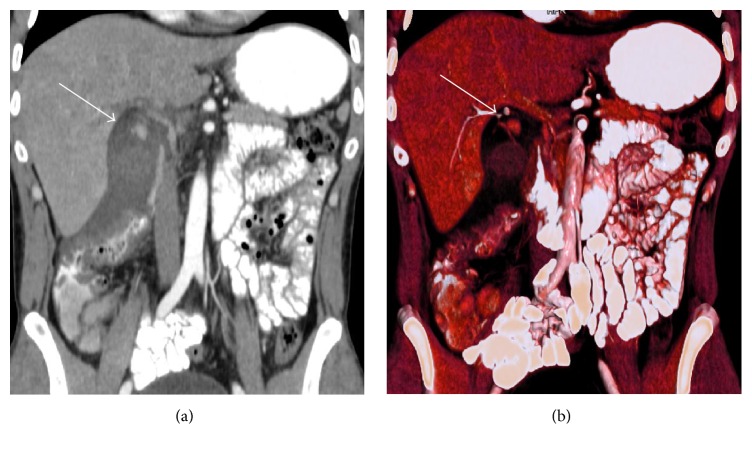
Coronal (a) and 3D reformatted images (b) of the patient. Arrows are indicating small cystic artery pseudoaneurysm.

**Figure 2:**
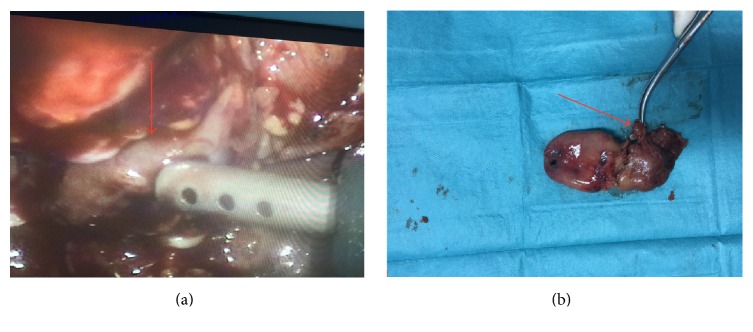
Pre- (a) and postoperative images (b). Arrows demonstrated cystic artery pseudoaneurysms.

36-year-old male patient was admitted to our surgery department with a right upper quadrant pain that started one day ago. He was afebrile, anicteric, and hemodynamically stable. His medical history was unremarkable. He did not use any medication. On physical examination, mild tenderness in right upper quadrant was noted. His laboratory test showed increased leukocytes (19 700/mm^3^) and C-reactive protein (CRP 7.9 mg/dL). His liver function tests and coagulation parameters were in normal limits. In light of these findings, first diagnosis that came into mind was acute cholecystitis. Ultrasound (USG) and contrast enhanced computed tomography (CT) were performed to confirm our prediagnosis. USG revealed cholelithiasis, thickening of gallbladder wall, and abundant amount of pericholecystic fluid that extend to the right paracolic area. On CT examination, in addition to findings of USG, small cystic artery pseudoaneurysm was identified (Figure 1). Since there was no clinical and radiological evidence of rupture and patient was hemodynamically stable, transcatheter embolization was not performed and laparoscopic cholecystectomy operation including pseudoaneurysm excision was planned. The patient had standard preoperative checks. Our operation was performed under general anesthesia. At operation, we put our first trocar through the umbilicus for induce pneumoperitoneum and after that we utilized standard 4-port laparoscopic cholecystectomy approach. Gall bladder was distended and inflamed. Very small cystic artery pseudoaneurysm was identified (Figure 2). Cystic duct and cystic artery were clipped and the gallbladder was dissected off the liver bed by standard technique. After achieving hemostasis control, suction drain was placed into the subhepatic space and operation was terminated.

## 3. Discussion

We herein reported a unique case of laparoscopic management of unruptured cystic artery pseudoaneurysm, which was presumedly due to acute cholecystitis. Most of the cases in literature are the complications of open or laparoscopic cholecystectomy [1]. To our knowledge, there are fewer than 30 cases that, associated with cholecystitis, were identified and also most of these cases were ruptured pseudoaneurysm [3]. However there were only few reports of incidentally diagnosed unruptured pseudoaneurysms and by our knowledge ours is the third one that managed with laparoscopic approach [2, 3]. The common symptoms of ruptured pseudoaneurysms included upper abdominal pain, jaundice, anemia, melena, and hematemesis [4]. USG, CT, and angiography are feasible tools for diagnosis of cystic artery pseudoaneurysms [3]. USG has ability to identify potential aneurysms but it may not detect small aneurysm like in our case. Although celiac or selective hepatic arteriography is the gold standard method for diagnosis, contrast enhanced CT is the best noninvasive method especially in emergency situations like acute abdomen or bleeding [3, 5]. According to most of the papers in current literature, when the aneurysms associated with cholecystitis, ligation of the cystic artery with open cholecystectomy is the main choice for treatment because of the high risk of aneurysmal rupture. However opposed to this aspect, there are some reports, which indicated that laparoscopic management is feasible and safe method for these patients [1]. In hemodynamically instable patients with ruptured pseudoaneurysms, angiographic embolization might be performed prior to definitive surgery [6].

In conclusion, in our case, preoperative use and careful evaluation of radiological methods were enabling us to detect cystic artery pseudoaneurysms. Due to high risk of rupture, surgeons have evaded laparoscopic approach to acute cholecystitis, which accompanied cystic artery pseudoaneurysm. However herein, we proved that laparoscopic management of cystic artery pseudoaneurysm with simultaneous cholecystectomy is feasible and reliable method in the hands of surgeons who have advanced laparoscopic skills.

## References

[1] Baker K. S., Tisnado J., Cho S.-R., Beachley M. C. (1987). Splanchnic artery aneurysms and pseudoaneurysms: transcatheter embolization. *Radiology*.

[2] Loizides S., Ali A., Newton R., Singh K. K. (2015). Laparoscopic management of a cystic artery pseudoaneurysm in a patient with calculus cholecystitis. *International Journal of Surgery Case Reports*.

[3] Machida H., Ueno E., Shiozawa S. (2008). Unruptured pseudoaneurysm of the cystic artery with acute calculous cholecystitis incidentally detected by computed tomography. *Radiation Medicine—Medical Imaging and Radiation Oncology*.

[4] Maeda A., Kunou T., Saeki S. (2002). Pseudoaneurysm of the cystic artery with hemobilia treated by arterial embolization and elective cholecystectomy. *Journal of Hepato-Biliary-Pancreatic Surgery*.

[5] Delgadillo X., Berney T., De Perrot M., Didier D., Morel P. (1999). Successful treatment of a pseudoaneurysm of the cystic artery with microcoil embolization. *Journal of Vascular and Interventional Radiology*.

[6] Mullen R., Suttie S. A., Bhat R., Evgenikos N., Yalamarthi S., McBride K. D. (2009). Microcoil embolisation of mycotic cystic artery pseudoaneurysm: a viable option in high-risk patients. *CardioVascular and Interventional Radiology*.

